# Mental health and quality of life in dialysis and transplant patients in Vietnam: a call for integrated care models

**DOI:** 10.3389/fpsyt.2025.1570138

**Published:** 2025-05-06

**Authors:** An Minh Nguyen, Long Hoang Vo

**Affiliations:** ^1^ Department of Surgical Nursing, Hanoi Medical College, Hanoi, Vietnam; ^2^ Department of Urology, Saint Paul General Hospital, Hanoi, Vietnam; ^3^ Department of Surgery, Thai Binh University of Medicine and Pharmacy, Thai Binh, Vietnam; ^4^ Department of Science, Technology, Communication and International Cooperation, E Hospital, Hanoi, Vietnam

**Keywords:** end-stage renal disease (ESRD), mental health disparities, integrated care models, public mental health, resource-limited settings

## Introduction

Chronic kidney disease represents a significant global health burden, affecting over 850 million individuals worldwide (1, 2). Among these, the progression to end-stage renal disease (ESRD) necessitates renal replacement therapies, including dialysis or kidney transplantation, to sustain life. While these treatments address the physiological needs of kidney failure, the psychological toll is profound (3–7). Studies report that approximately 30–40% of dialysis patients suffer from clinically significant depression and anxiety, underscoring the substantial mental health burden associated with ESRD (8–10). In Vietnam and similar resource-constrained settings, financial stress, limited psychological care, and social stigma further heighten patients’ vulnerability (11, 12).

Despite evidence highlighting the interconnectedness of physical symptoms and HRQOL in ESRD (13), healthcare models remain siloed, particularly in low- and middle-income countries (LMICs). Current approaches often neglect the multidimensional burden of symptoms, from pain and fatigue to emotional distress, that define patients’ lived experiences. Lessons from integrated mental health-primary care models demonstrate that collaborative frameworks can improve outcomes for chronic conditions, though their adaptation to nephrology care, especially in LMICs, remains underexplored (14). This viewpoint critiques this fragmentation through a Vietnamese context, advocating for integrated care models that address both nephrological and mental health needs. By synthesizing global insights (e.g., symptom assessment frameworks) with local realities, we propose scalable strategies to bridge this gap, emphasizing interdisciplinary collaboration and patient-centered outcomes.

## Impact on mental health and quality of life

### Mental health implications

Patients receiving dialysis or kidney transplants face intertwined physical and psychological challenges. Clinical evidence reveals disproportionately high rates of depression, anxiety, and sleep disturbances in this population, with pooled prevalence estimates reaching ~50% across studies (15–17). These figures, however, reflect methodological variations in assessment tools (e.g., PHQ-9 vs. BDI) and cultural adaptations across study populations. In resource-limited settings like Vietnam, these conditions are further exacerbated by financial strain on patients and families (18), including costs of lifelong dialysis/post-transplant medications and inadequate insurance coverage (19). This economic burden perpetuates a vicious cycle of mental health risks and caregiver burnout.

Cultural factors also play a pivotal role. Social stigma associated with chronic illness and mental health issues can deter patients from seeking psychological support, perpetuating feelings of isolation and distress (20). In Vietnam, traditional beliefs regarding illness causation and healing may conflict with modern mental health approaches, creating additional barriers to accessing care.

### Quality of life

ESRD severely limits daily functioning. Dialysis schedules and post-transplant regimens often impose rigid constraints on daily life, though the degree of impact may vary by individual circumstances and treatment modalities. Fatigue, physical discomfort, and dietary restrictions further diminish quality of life, making routine tasks burdensome (21–23).

Interpersonal relationships are similarly affected. While strong family support can be a protective factor, it may also become a source of tension if caregiving demands are overwhelming or inadequately shared (24, 25). Marital and familial dynamics often undergo substantial strain, influencing both mental health outcomes and overall well-being (26). Addressing these multifaceted impacts requires a holistic approach that integrates psychological and social dimensions into routine nephrology care.

## Barriers to integrated mental and physical care in Vietnam

### Healthcare system constraints

Vietnam’s healthcare infrastructure struggles with a critical shortage of healthcare professionals trained simultaneously in nephrology and psychiatry. The specialized expertise required to address both the physical and psychological aspects of chronic kidney disease is fragmented, with nephrology and mental health services operating as separate, siloed disciplines. This division hampers comprehensive care and prevents the holistic management of patients’ complex needs.

Additionally, a lack of interdisciplinary collaboration further exacerbates the disconnect. Referral pathways between nephrologists and mental health practitioners are often unclear or non-existent, leaving patients without timely psychological support. This systemic fragmentation poses significant barriers to the integration of care that addresses both mind and body.

### Financial barriers

Economic challenges compound the difficulties faced by renal patients in Vietnam (18, 27, 28). The cost of dialysis, transplantation, and long-term immunosuppressive therapies is substantial, placing enormous financial strain on households (27–29). Psychological services, when available, are often excluded from standard health insurance plans, making them inaccessible to most patients.

The absence of comprehensive insurance coverage for mental health care creates a significant gap in treatment. Without adequate financial support, both patients and caregivers endure chronic stress, contributing to a vicious cycle of deteriorating mental and physical health outcomes. Addressing these financial inequities is critical to building a sustainable, integrated model of care that prioritizes both mental health and nephrological outcomes.

## Proposed model for integrated care

**Figure 1:**
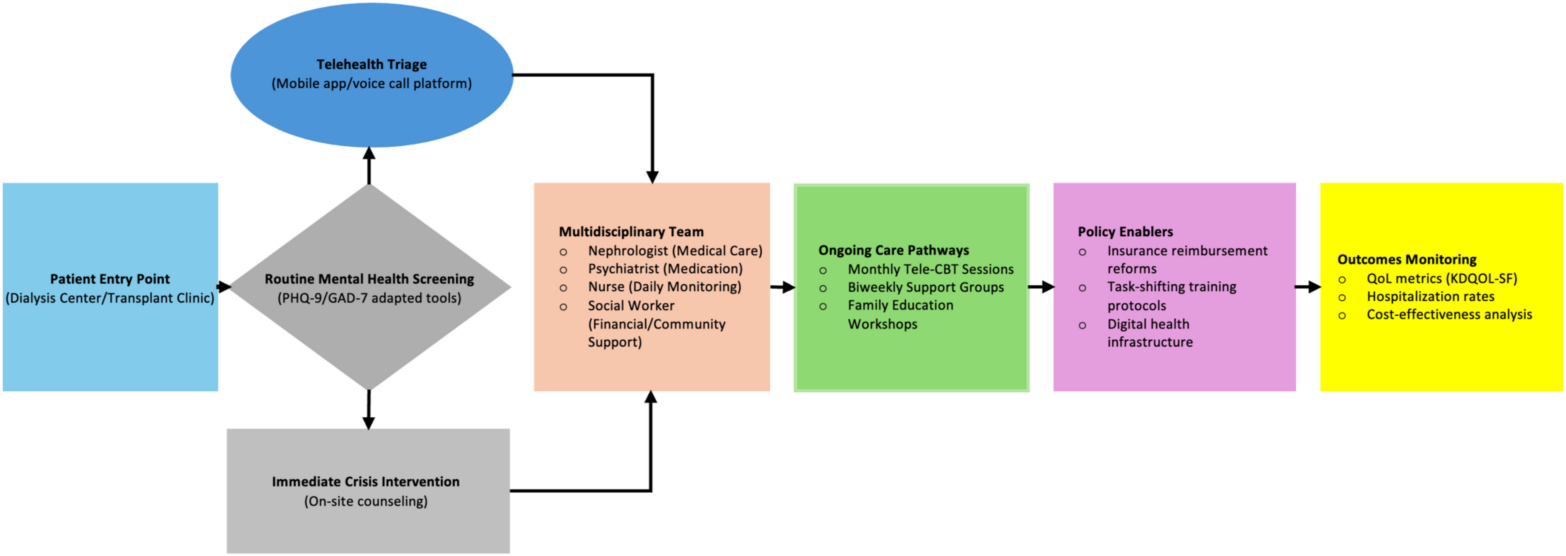
Integrated ESRD care framework for LMICs *(Vietnam use case).*.

Addressing the dual burdens of ESRD requires a holistic and patient-centered approach that integrates mental health with routine nephrology care. A multidisciplinary care model that encompasses nephrologists, mental health professionals, and social workers is essential to provide comprehensive support. First, embedding psychological counseling and mental health screening as routine components of dialysis and transplant care is imperative. Mental health interventions should be standardized, ensuring that patients receive regular assessments for depression, anxiety, and other psychosocial challenges (30). Specialized mental health professionals should collaborate closely with nephrology teams to develop personalized care plans. Second, establishing dedicated mental health services within dialysis centers can reduce stigma and improve accessibility. Group therapy sessions and peer support initiatives can foster a supportive community and normalize mental health discussions. Third, leveraging telehealth solutions offers a cost-effective strategy to overcome geographical and resource limitations (31). Remote counseling and psychological support, delivered through mobile platforms, can extend mental health services to rural areas and underserved populations. These technologies should be tailored to the socio-economic context of Vietnam to ensure affordability and usability. Integrated ESRD care model for Vietnam was illustrated in **Figure 1**.

Consistent with evidence from Butler et al. (2008) (14), successful integration requires addressing both clinical workflows (e.g., shared screening protocols) and systemic barriers (e.g., reimbursement structures), a dual focus our model adapts for Vietnam’s renal care context. By adopting these three strategies, Vietnam’s healthcare system can build a scalable, integrated model addressing both physical and mental health needs, though three implementation challenges require attention. First, workforce training barriers could be addressed through modular programs adapting WHO mhGAP curricula. Second, infrastructure limitations may be overcome by phased implementation prioritizing high-volume centers. Finally, cultural resistance to telehealth could be mitigated through blended care models to increase patient engagement. While this model requires local adaptation, its evidence-based solutions for training, infrastructure, and technology adoption provide a viable blueprint for LMICs seeking comprehensive renal care.

## Policy implications and global relevance

The health policy landscape for managing ESRD and its associated mental health burden requires a paradigm shift toward a more inclusive, comprehensive, and cost-effective approach. The disproportionate impact of ESRD on vulnerable populations in low- and middle-income countries (LMICs) demands the adoption of integrated care models that harmonize both physical and mental health services. Similar trends are observed in other LMICs, where ESRD patients face comparable mental health disparities due to financial constraints and siloed care systems (32–35). This highlights the urgent need for scalable, integrated models across resource-limited settings. Effective, evidence-based policy reforms must prioritize holistic healthcare systems that encompass not only the medical management of ESRD but also address the psychological and social well-being of patients. The mixed results of integration levels on outcomes caution against one-size-fits-all models, instead advocating for context-specific adaptations (14), a principle central to our Vietnam-focused recommendations. These policies should be tailored to the unique financial, cultural, and infrastructural challenges of LMICs, ensuring that mental health support is not relegated to a secondary concern but is woven into the core of renal care.

In particular, the implementation of integrated care models that combine multidisciplinary healthcare teams, psychological counseling, and community-based support networks represents a critical avenue for improving patient outcomes. Such policies should advocate for the inclusion of mental health services in standard treatment protocols for dialysis and transplant patients, and promote strategies that reduce the economic burden on families through improved access to insurance and subsidized care. Furthermore, robust monitoring and evaluation frameworks must be established to assess the effectiveness and scalability of these interventions within resource-limited settings.

Given the global reach of ESRD, key international health organizations, such as the World Health Organization (WHO), the World Kidney Fund (WKF), the International Society of Nephrology (ISN), and the Global Kidney Health Alliance (GKHA), are well-positioned to lead coordinated efforts. These bodies, in collaboration with other relevant regulatory agencies, should prioritize the combined challenge of ESRD and its mental health impact in global health agendas. A collective, worldwide initiative is essential to design policies that address both the medical and psychological needs of ESRD patients, recognizing the profound effect mental health has on their quality of life. By advancing integrated care models, these organizations can provide a framework for national health systems, promoting cross-border cooperation and the development of equitable and sustainable healthcare solutions.

## Conclusion

In conclusion, the mental health challenges faced by patients with ESRD are profound, often exacerbated by the physical toll of dialysis and transplantation, compounded by socio-economic constraints, and reinforced by cultural stigma. Vietnam, as a representative of many LMICs, exemplifies the urgent need for integrated care models that address both the physical and psychological needs of ESRD patients. The current healthcare system’s siloed approach, which separates nephrology from mental health care, leaves patients vulnerable to significant, unmet psychological distress.

The evidence presented in this editorial underscores the necessity of a multidisciplinary approach to care, one that brings together nephrologists, psychologists, social workers, and community resources to deliver a holistic care model. Such an integrated approach is not only a moral imperative but a practical one, especially in settings with limited resources. The proposed strategies, including routine mental health screening, embedding psychological services in dialysis centers, and leveraging telehealth solutions, offer a scalable framework that can be adapted across LMICs, paving the way for global health initiatives aimed at improving the quality of life for ESRD patients.

Policy reform is critical to transforming the landscape of ESRD care. Governments and international organizations must prioritize the integration of mental health into renal care protocols and advocate for financial mechanisms that ensure mental health services are accessible and affordable. With a global perspective, this call for action extends beyond Vietnam, urging a collective commitment to sustainable, patient-centered care that addresses the dual burdens of physical and mental health in ESRD patients worldwide.

As we look to the future, the adoption of these integrated models of care, supported by robust policies and international cooperation, holds the potential to significantly improve the well-being of millions of patients facing kidney failure. By embracing a comprehensive approach, we can ensure that ESRD patients receive not only life-sustaining treatment but also the mental health support they need to lead fulfilling lives.
